# Residential greenness and atopic dermatitis in Japanese children: Findings from the TMM BirThree cohort study

**DOI:** 10.1111/pai.70338

**Published:** 2026-04-06

**Authors:** Ami Uematsu, Hisashi Ohseto, Masatsugu Orui, Zheng Xian, Yuta Takahashi, Geng Chen, Keiko Murakami, Mami Ishikuro, Aoi Noda, Genki Shinoda, Taku Obara, Tomoki Nakaya, Shinichi Kuriyama

**Affiliations:** ^1^ Graduate School of Medicine Tohoku University Sendai Miyagi Japan; ^2^ International Research Institute of Disaster Science Tohoku University Sendai Miyagi Japan; ^3^ Tohoku Medical Megabank Organization Tohoku University Sendai Miyagi Japan; ^4^ Department of Frontier Sciences for Advanced Environment, Graduate School of Environmental Studies Tohoku University Sendai Miyagi Japan; ^5^ Graduate School of Medicine The University of Tokyo Tokyo Japan; ^6^ Tohoku University Hospital Tohoku University Sendai Miyagi Japan; ^7^ Department of Earth Science, Graduate School of Science Tohoku University Sendai Miyagi Japan

**Keywords:** allergy, atopic dermatitis, children, Japan, normalized difference vegetation index, residential greenness

## Abstract

**Background:**

Atopic dermatitis (AD) is a chronic inflammatory skin disease that primarily develops during childhood. Residential greenness has been reported to be associated with AD onset. However, findings are inconsistent and may not apply to Japanese settings because vegetation and residential environments vary regionally. Therefore, this study aimed to examine early‐life exposure to residential greenness and subsequent AD development in Japanese children.

**Methods:**

We analyzed data from 14,932 children in the Tohoku Medical Megabank Project Birth and Three‐Generation Cohort Study. The exposure was residential greenness during the first 6 months of life, quantified using the normalized difference vegetation index (NDVI) within 250‐, 500‐, and 1000‐m buffers around each birth postal code and categorized into tertiles (low [reference], moderate, high). The outcome was AD incidence between 6 months and 5 years of age, assessed via maternal reporting. Adjusted risk ratios (RRs) and 95% confidence intervals (CIs) were estimated using modified Poisson regression. Analyses were stratified by urbanization levels, child's sex, and parental history of allergic diseases.

**Results:**

A significant protective association was observed among children in the high NDVI tertile within the 250‐m buffer (adjusted RR: 0.80, 95% CI: 0.67–0.95), whereas the moderate tertile was not significant (adjusted RR: 0.94, 95% CI: 0.83–1.06). A similar pattern was observed at 500 m. Stratification showed similar associations across urbanization levels, which were stronger in boys and observed only among children without parental allergic history.

**Conclusion:**

Early‐life exposure to residential greenness was associated with a decreased risk of AD in Japanese children.

**Key message**

This study is the first in Japan to investigate the association between residential greenness (assessed using the normalized difference vegetation index) and the development of atopic dermatitis in children. Early life exposure to residential greenness was protectively associated with the development of atopic dermatitis for up to 5 years of age.

## 1 INTRODUCTION

Atopic dermatitis (AD) is a chronic inflammatory skin disease characterized by intense itching and eczematous lesions.^1^, ^2^ It typically develops before the age of 5 years, with a prevalence of approximately 15%–20% among children globally.^1^, ^3^ Although most children experience remission by adulthood, AD persists into adulthood in approximately 10%–30% of cases.^1^ It adversely affects patients' quality of life and increases healthcare costs. Additionally, parents of young children with AD are often burdened by sleep deprivation and the suffering of their children.^2^, ^4^

The pathogenesis of AD involves complex interactions between genetic and environmental factors, characterized by cutaneous barrier dysfunction, immune dysregulation associated with a Th2 response, and microbial imbalance.^2^, ^5^ Early‐life environmental exposures play a critical role in immune development.^6^, ^7^, ^8^ This period coincides with the maturation of the infant's immune system after maternal antibodies wane.^9^ Accordingly, identifying early‐life determinants of pathogenic mechanisms crucial for immune development has garnered increasing research interest.

Greenness refers to vegetation such as trees, grass, and other plants, encompassing both planned areas (e.g., parks and street trees) and unplanned natural regions.^10^ Recently, exposure to greenness has been reported to have beneficial effects on various health outcomes, including obesity, type II diabetes, cardiovascular disease, mental health, and perinatal outcomes.^11^, ^12^, ^13^, ^14^, ^15^ Additionally, greenness is considered to promote health by reducing exposure to noise and heat, removing air pollutants, encouraging physical activity and social interaction, alleviating psychophysiological stress, and providing biodiverse natural environments.^16^, ^17^, ^18^, ^19^ In contrast, its effects appear more complex regarding allergic diseases. An increase in greenness may reduce the incidence of allergic symptoms through its effects on immunity and the microbiota; however, greenness may also be a direct source of allergens, aggravating allergic reactions.^19^, ^20^

Previous research has examined the association between greenness from the prenatal period through childhood and AD in children^21^, ^22^, ^23^, ^24^, ^25^, ^26^, ^27^, ^28^, ^29^; however, the findings have been inconsistent. Some studies suggested that greenness has a protective effect,^21^, ^22^, ^23^ whereas others reported an increased risk,^24^, ^25^ and some even found no significant association.^26^, ^27^, ^28^, ^29^

Because the composition of vegetation and residential environments differs across countries and regions, geographic‐specific research is needed. However, to the best of our knowledge, no studies in Japan have investigated the association between greenness and AD. Moreover, the effects of greenness have been reported to differ between urban and non‐urban areas.^30^ In addition, AD has been reported to be more prevalent in boys during early childhood and among children with a parental history of allergic diseases.^1^, ^31^ Therefore, this study aimed to investigate the association between early‐life exposure to residential greenness and the subsequent development of AD in Japanese children, and to examine whether the association differs by urbanization level, child's sex, and parental history of allergic diseases.

## 2 METHODS

### 2.1 Study population

The present study utilized data from the Tohoku Medical Megabank Project Birth and Three‐Generation Cohort Study (TMM BirThree Cohort Study). Detailed information on the TMM BirThree Cohort Study has been previously published.^32^ Pregnant women and their family members were recruited between July 2013 and March 2017 from obstetric clinics and hospitals in Miyagi and Iwate prefectures, Japan. In total, 23,730 mother–child pairs participated in the study.

**FIGURE 1:**
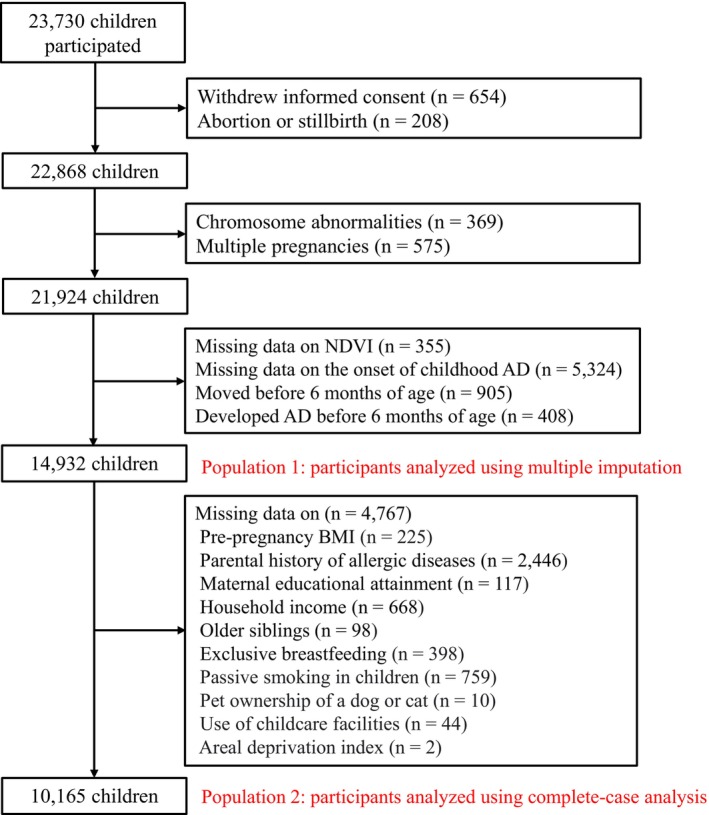
Flow chart of participant selection. The flowchart shows the exclusion criteria and the total, excluded, and included numbers of participants. AD, atopic dermatitis; BMI, body mass index; NDVI, normalized difference vegetation index.

In the present study, among the 22,868 participants after excluding those with withdrawn informed consent and cases of abortion or stillbirth, 16,961 (74.2%) returned at least one questionnaire between ages 1 and 5 years and were eligible for assessment of AD. Children with withdrawn informed consent (*n* = 654), cases of abortion or stillbirth (*n* = 208), those with chromosomal abnormalities (*n* = 369), or mothers with multiple pregnancies (*n* = 575) were excluded. In addition, children with missing residential greenness data (*n* = 355), those for whom no questionnaire was ever returned (*n* = 5324), those who moved residence within the first 6 months of life (*n* = 905), and those who developed AD within the first 6 months (*n* = 408) were also excluded. Children who developed AD before 6 months of age were excluded, as early‐onset disease is likely to be strongly influenced by prenatal and immediate postnatal factors. Participants who moved within the first 6 months of life were also excluded because early relocation could lead to exposure misclassification in the assessment of greenness. After these exclusions, data from 14,932 children were included in the present study (Figure 1).

### 2.2 Exposure to greenness

Residential greenness was assessed using the normalized difference vegetation index (NDVI), which is one of the most commonly used satellite‐based measures of vegetation,^33^ calculated from Landsat 8 imagery at a 30‐m spatial resolution via Google Earth Engine.^34^, ^35^ NDVI was derived from the near‐infrared and red spectral bands, with values ranging from −1 to +1. Values ≤0.1 typically indicate barren areas, such as rock, sand, water, or snow. Moderate values (0.2–0.3) are generally associated with shrubs and grasslands, whereas higher values (0.6–0.8) indicate denser vegetation cover, commonly in forest areas.^35^ Given that Japan's urbanization process was largely completed, with minimal year‐to‐year variation in urban green spaces, we assumed that urban green space is relatively stable over time^36^ and used the 2015 NDVI values as a proxy, corresponding to the midpoint of participant recruitment (2013–2017). To reduce the influence of seasonal variation, all available remote sensing images from January, April, July, and October 2015 were used. Cloud and cloud‐shadow contamination were removed using masking algorithms, and monthly composites were generated to minimize data gaps. For each buffer zone, the annual mean NDVI was calculated as an indicator of residential greenness exposure. NDVI values were linked to the centroid coordinates of postal code areas reported at the time of the child's enrollment to the cohort. To assess greenness exposure at different spatial scales, circular buffers with radii of 250 m, 500 m, and 1000 m were created around each centroid. Based on tertiles of the mean NDVI within each buffer, exposure was classified as low, moderate, or high.

### 2.3 Development of AD

The development of AD in children was assessed using maternal responses to self‐reported questionnaires. The questionnaire included the following question: “Since the last survey, has a physician diagnosed your child with atopic dermatitis?” Mothers indicated the diagnosis by checking a box. For this study, questionnaires from each survey were conducted when the child was at 12, 24, 36, 42, 48, and 60 months of age. Follow‐up assessments were generally conducted at 12‐month intervals, but the assessment at 42 months was conducted concurrently to coincide with the timing of routine toddler health checkups in Japan. Children who were reported to have developed AD at least once during these assessments were classified as having AD.

### 2.4 Covariates

The analyses were adjusted for the following covariates, selected based on previous studies examining the association between greenness and AD^21^, ^22^, ^23^, ^24^, ^25^, ^26^, ^27^, ^28^, ^29^: maternal age at delivery (<25, 25–29, 30–34, and ≥35 years), maternal pre‐pregnancy body mass index (BMI; <18.5, 18.5–24.9, and ≥25.0 kg/m^2^), maternal history of allergic diseases (yes/no), paternal history of allergic diseases (yes/no), maternal educational attainment (high school or lower, junior or vocational college, and university or higher), household income (<4.00, 4.00–5.99, and ≥6.00 million Japanese yen/year), child's sex (boy or girl), season of birth (winter: December–February; spring: March–May; summer: June–August; autumn: September–November), presence of older siblings (yes/no), exclusive breastfeeding (yes/no), passive smoking in children (yes/no), pet ownership of dogs or cats (yes/no), and use of childcare facilities (yes/no). Parental history of allergic diseases was defined based on self‐reported physician‐diagnosed bronchial asthma, AD, food allergy, allergic conjunctivitis, or allergic rhinitis. Data on maternal height, pre‐pregnancy weight, and the child's sex were obtained from medical records, whereas all other covariates were obtained via self‐reported questionnaires by mothers. Information on exclusive breastfeeding, passive smoking in children, pet ownership, and use of childcare facilities was collected from questionnaires completed until the child was 6 months of age.

Additionally, the following geographic variables were included as covariates: air pollution (nitrogen dioxide [NO_2_] and particulate matter ≤2.5 μm [PM_2.5_]), the areal deprivation index (ADI), and urbanization level. Air pollution was assessed using the annual average concentrations of NO_2_ and PM_2.5_ in 2015, predicted by universal kriging models. Potential predictors for the models included traffic intensity, population, land use, elevation, and geographic coordinates. The models were estimated on 100‐m predicted points, and postal‐code–level values were calculated as the mean of the predicted values within each postal code area.^15^ The ADI, calculated using data from the 2015 Japan Census,^37^ is a composite indicator defined as the weighted sum of the proportions of socially and economically disadvantaged groups, including older couple households, older single households, rental households, single‐mother households, sales and service workers, agricultural workers, blue‐collar workers, and unemployed individuals. ADI values at the postal code level were derived through proportionally allocating census‐level ADI values to the corresponding areas. The urbanization level was defined based on population density and distance from the densely inhabited district, a geographical unit representing urban areas in Japan, and it was categorized into six levels: metropolitan areas, large cities, accessible small towns, remote small towns, accessible rural settlements, and remote rural settlements. These categories were subsequently reclassified into “metropolitan” (including the densely inhabited districts of Sendai City, the capital and largest city of Miyagi Prefecture) and “non‐metropolitan” (all other areas), which were used in stratified analyses.^38^

### 2.5 Statistical analysis

Participant characteristics were summarized across tertiles of greenness within a 250‐m buffer. To examine associations between greenness within 250‐, 500‐, and 1000‐m buffers and AD, modified Poisson regression models were applied, with NDVI tertiles defined separately for each buffer, using the lowest NDVI tertile as the reference. These models were chosen, rather than a time‐to‐event analysis, to retain participants with partial follow‐up and use all available data up to age 5 years. Results were expressed as risk ratios (RRs) with 95% confidence intervals (CIs). Both crude and adjusted models were fitted. Adjusted models included the following covariates: maternal age at delivery, maternal pre‐pregnancy BMI, maternal history of allergic diseases, paternal history of allergic diseases, maternal educational attainment, household income, child's sex, season of birth, presence of older siblings, exclusive breastfeeding, passive smoking in children, pet ownership of dogs or cats, use of childcare facilities, NO_2_ and PM_2.5_ levels, ADI, and urbanization level. Missing data were handled using multiple imputation by chained equations (Population 1; Figure 1). The imputation model included all variables from the main analysis, and 30 imputed datasets were created using the *mice* package in R.^39^ To evaluate robustness, complete case analysis was also conducted for sensitivity (Population 2; Figure 1). In addition, we performed a sensitivity analysis applying common tertile cutoffs based on the NDVI distribution within the 250‐m buffer to the 500‐m and 1000‐m buffers to facilitate comparison across buffer sizes.

Stratified analyses were conducted according to urbanization level (metropolitan/non‐metropolitan), child's sex (boy/girl), and parental history of allergic diseases (with/without); “with” included children with either one or both parents having a history of allergic disease. The interaction terms between residential greenness and each of these variables were included in the models, and *p* values for interaction were calculated. The stratification considered potential differences in green environment characteristics, sex differences, and gene–environment interactions. In addition, as a sensitivity analysis, we explored potential heterogeneity within the non‐metropolitan category using more detailed urbanization subcategories, including large cities and accessible small towns (combined due to the small number of large cities), remote small towns, accessible rural settlements, and remote rural settlements.

All statistical analyses were conducted using R (version 4.1.2; R Foundation, Vienna, Austria). Two‐sided *p* values <.05 were considered statistically significant.

An artificial intelligence tool (ChatGPT, OpenAI) was used to assist with grammar and language editing. All intellectual contributions and final revisions were performed by the authors.

### 2.6 Ethics approval and consent to participate

The TMM BirThree Cohort Study protocol was reviewed and approved by the Ethics Committee of Tohoku University Tohoku Medical Megabank Organization (2013–1–103‐1; latest revision 2024–4‐021). All procedures were conducted in accordance with the Declaration of Helsinki. All participants provided informed consent at enrollment. For those who were unable to fully understand the study protocol at any age, informed consent was obtained from their guardians with approval from the Ethics Committee.

## 3 RESULTS

Table 1 shows the baseline characteristics of participants according to tertiles of NDVI within a 250‐m buffer. The distribution of NDVI within a 250‐m buffer ranged from 0.043 to 0.372, with tertiles defined as low (<0.097), moderate (0.097–0.136), and high (≥0.136). The distributions of NDVI at the 500‐ and 1000‐m buffers ranged from 0.039 to 0.378 and from 0.052 to 0.402, respectively. Children living in areas with high NDVI tended to have mothers who were younger at delivery and had higher pre‐pregnancy BMI, lower educational attainment, and lower household income, as well as parents without a history of allergic diseases. They also tended to have older siblings, were not exclusively breastfed for 6 months, were exposed to passive smoking, and lived with pets. The number of children who developed AD by 5 years of age was 535 (10.7%) in the low NDVI group, 527 (10.6%) in the moderate group, and 475 (9.5%) in the high group.

**TABLE 1:** Participants' characteristics.

	Total	Tertiles of NDVI within a 250 m buffer			*p*‐value
		Low	Moderate	High	
	*n* = 14,932	*n* = 4993	*n* = 4965	*n* = 4974	
Development of atopic dermatitis, *n* (%)	1537 (10.3)	535 (10.7)	527 (10.6)	475 (9.5)	.106
Maternal age at delivery (years), *n* (%)					
<25	966 (6.5)	263 (5.3)	327 (6.6)	376 (7.6)	<.001
25–29	3684 (24.7)	1183 (23.7)	1203 (24.2)	1298 (26.1)	
30–34	5564 (37.3)	1861 (37.3)	1897 (38.2)	1806 (36.3)	
≥35	4718 (31.6)	1686 (33.8)	1538 (31.0)	1494 (30.0)	
Pre‐pregnancy BMI (kg/m^2^), *n* (%)					
<18.5	1946 (13.0)	693 (13.9)	656 (13.2)	597 (12.0)	<.001
18.5–24.9	10,889 (72.9)	3688 (73.9)	3650 (73.5)	3551 (71.4)	
≥25.0	1872 (12.5)	536 (10.7)	589 (11.9)	747 (15.0)	
Maternal history of allergic diseases, *n* (%)					
No	7581 (50.8)	2557 (51.2)	2501 (50.4)	2523 (50.7)	.001
Yes	4869 (32.6)	1681 (33.7)	1636 (33.0)	1552 (31.2)	
Paternal history of allergic diseases, *n* (%)					
No	9597 (64.3)	3247 (65.0)	3185 (64.1)	3165 (63.6)	.002
Yes	2853 (19.1)	991 (19.8)	952 (19.2)	910 (18.3)	
Maternal educational attainment, *n* (%)					
High school or lower	4074 (27.3)	1111 (22.3)	1364 (27.5)	1599 (32.1)	<.001
Junior or vocational college	4794 (32.1)	1658 (33.2)	1560 (31.4)	1576 (31.7)	
University or higher	3464 (23.2)	1431 (28.7)	1173 (23.6)	860 (17.3)	
Household income (million Japanese yen/year), *n* (%)					
<4.00	4909 (32.9)	1490 (29.8)	1652 (33.3)	1767 (35.5)	<.001
4.00–5.99	4619 (30.9)	1556 (31.2)	1622 (32.7)	1441 (29.0)	
≥6.00	4468 (29.9)	1683 (33.7)	1407 (28.3)	1378 (27.7)	
Child's sex, *n* (%)					
Boy	7698 (51.6)	2591 (51.9)	2511 (50.6)	2596 (52.2)	.229
Girl	7234 (48.4)	2402 (48.1)	2454 (49.4)	2378 (47.8)	
Season of birth, *n* (%)					
Winter [December–February]	3672 (24.6)	1206 (24.2)	1219 (24.6)	1247 (25.1)	.680
Spring [March–May]	3788 (25.4)	1278 (25.6)	1252 (25.2)	1258 (25.3)	
Summer [June–August]	3587 (24.0)	1175 (23.5)	1198 (24.1)	1214 (24.4)	
Autumn [September–November]	3885 (26.0)	1334 (26.7)	1296 (26.1)	1255 (25.2)	
Older siblings, *n* (%)					
No	7154 (47.9)	2709 (54.3)	2355 (47.4)	2090 (42.0)	<.001
Yes	7490 (50.2)	2186 (43.8)	2511 (50.6)	2793 (56.2)	
Exclusive breastfeeding, *n* (%)					
No	8930 (59.8)	2698 (54.0)	3001 (60.4)	3231 (65.0)	<.001
Yes	4878 (32.7)	1888 (37.8)	1605 (32.3)	1385 (27.8)	
Passive smoking in children, *n* (%)					
No	11,693 (78.3)	3974 (79.6)	3888 (78.3)	3831 (77.0)	.002
Yes	638 (4.3)	172 (3.4)	220 (4.4)	246 (4.9)	
Pet ownership of a dog or cat, *n* (%)					
No	9740 (65.2)	3448 (69.1)	3336 (67.2)	2956 (59.4)	<.001
Yes	3463 (23.2)	960 (19.2)	1041 (21.0)	1462 (29.4)	
Use of childcare facilities, *n* (%)					
No	11,280 (75.5)	3839 (76.9)	3726 (75.0)	3715 (74.7)	.045
Yes	1310 (8.8)	414 (8.3)	462 (9.3)	434 (8.7)	
Urbanization level, *n* (%)					
Metropolitan areas	7454 (49.9)	4126 (82.6)	2667 (53.7)	661 (13.3)	<.001
Large cities	7 (0.0)	2 (0.0)	1 (0.0)	4 (0.1)	
Accessible small towns	967 (6.5)	14 (0.3)	584 (11.8)	369 (7.4)	
Remote small towns	1574 (10.5)	464 (9.3)	854 (17.2)	256 (5.1)	
Accessible rural settlements	1438 (9.6)	155 (3.1)	424 (8.5)	859 (17.3)	
Remote rural settlements	3491 (23.4)	231 (4.6)	435 (8.8)	2825 (56.8)	
Areal deprivation index (mean (SD))	5.557 (0.892)	5.501 (0.786)	5.531 (0.923)	5.638 (0.952)	<.001
NO_2_, ppm (mean (SD))	0.008 (0.002)	0.010 (0.002)	0.008 (0.001)	0.007 (0.002)	<.001
PM_2.5_, μg/m^3^ (mean (SD))	10.942 (0.503)	11.229 (0.246)	10.985 (0.322)	10.612 (0.633)	<.001
NDVI (mean (SD))					
Within a 250‐m buffer	0.124 (0.050)	0.077 (0.012)	0.115 (0.010)	0.181 (0.040)	<.001
Within a 500‐m buffer	0.136 (0.053)	0.083 (0.012)	0.127 (0.012)	0.198 (0.039)	<.001
Within a 1000‐m buffer	0.149 (0.055)	0.093 (0.015)	0.141 (0.012)	0.214 (0.037)	<.001

*Note*: Data are presented as mean (standard deviation) for continuous variables and *n* (%) for categorical variables.

Abbreviations: BMI, body mass index; NDVI, normalized difference vegetation index; SD, standard deviation.

Table 2 shows the RRs and 95% CIs estimated from the modified Poisson regression models. In the adjusted models, for NDVI within a 250‐m buffer, the RRs were 0.94 (95% CI: 0.83–1.06) for the moderate group and 0.80 (95% CI: 0.67–0.95) for the high group. For the 500‐m buffer, the RRs were 0.88 (95% CI: 0.78–1.01) for moderate and 0.83 (95% CI: 0.69–0.99) for high. For the 1000‐m buffer, the RRs were 0.94 (95% CI: 0.83–1.08) for moderate and 0.91 (95% CI: 0.76–1.10) for high (upper panel of Table 2). Significant trends were observed for the 250‐m (*p* for trend = .014) and 500‐m (*p* = .031) buffers, but not for the 1000‐m buffer (*p* = .299). The complete case analysis produced similar results (lower panel of Table 2). In the sensitivity analysis applying the 250‐m buffer cutoffs (low <0.097, moderate 0.097–0.136, high ≥0.136) to the 500‐m and 1000‐m buffers, the results were generally consistent with the primary findings, with similar directions and magnitudes of associations observed across buffer sizes (Table S1).

**TABLE 2:** Association between residential greenness and atopic dermatitis in children.

Buffer	NDVI	Crude model			Adjusted model		
		RR (95% CI)	*p*‐value	*p* for trend	RR (95% CI)	*p*‐value	*p* for trend
Multiple imputation							
250 m	Low	Ref		.054	Ref		.**014**
	Moderate	0.99 (0.88–1.11)	.871		0.94 (0.83–1.06)	.318	
	High	0.89 (0.79–1.00)	.054		**0.80 (0.67–0.95)**	.**012**	
500 m	Low	Ref		.090	Ref		.**031**
	Moderate	0.94 (0.83–1.05)	.251		0.88 (0.78–1.01)	.061	
	High	0.90 (0.81–1.02)	.090		**0.83 (0.69–0.99)**	.**040**	
1000 m	Low	Ref		.273	Ref		.299
	Moderate	0.97 (0.87–1.09)	.633		0.94 (0.83–1.08)	.386	
	High	0.94 (0.83–1.05)	.273		0.91 (0.76–1.10)	.319	
Complete case							
250 m	Low	Ref		.107	Ref		.**003**
	Moderate	0.97 (0.84–1.11)	.615		0.89 (0.77–1.04)	.136	
	High	0.89 (0.78–1.03)	.108		**0.74 (0.60–0.90)**	.**003**	
500 m	Low	Ref		.291	Ref		.**027**
	Moderate	0.97 (0.84–1.11)	.614		0.89 (0.77–1.04)	.156	
	High	0.93 (0.81–1.07)	.291		**0.79 (0.64–0.97)**	.**028**	
1000 m	Low	Ref		.309	Ref		.**023**
	Moderate	1.00 (0.88–1.15)	.969		0.91 (0.78–1.07)	.252	
	High	0.93 (0.81–1.07)	.309		**0.77 (0.61–0.96)**	.**020**	

*Note*: Adjusted model: Adjusted for maternal age at delivery, maternal pre‐pregnancy body mass index, maternal history of allergic diseases, paternal history of allergic diseases, maternal educational attainment, household income, child's sex, season of birth, presence of older siblings, exclusive breastfeeding, passive smoking in children, pet ownership of dogs or cats, use of childcare facilities, NO_2_, PM_2.5_, areal deprivation index, urbanization level. Bold: *p* < .05.

Abbreviations: CI, confidence interval; NDVI, normalized difference vegetation index; RR, risk ratio.

Table 3 presents the results of the stratified analyses. Overall, the protective association between residential greenness and AD was the strongest at small buffer sizes. In analyses stratified by urbanization level, the associations were consistent between metropolitan and non‐metropolitan areas. Although a statistically significant interaction was observed for the high group within the 1000‐m buffer, this finding was limited to the 1000‐m buffer only, and the point estimates were largely similar between the two areas. In sensitivity analyses with further stratification of non‐metropolitan areas, the results were largely consistent; however, a significantly increased risk was observed for the 1000‐m buffer in large cities and accessible small towns (Table S2), although the sample size in this stratum was relatively small (*N* < 1000). In analyses stratified by the child's sex, the protective association was more evident among boys, with similar but weaker patterns among girls. However, no statistically significant interaction was observed. When stratified by parental history of allergic diseases, the protective association was observed only among children without a parental history of allergic diseases, with statistically significant interactions observed for the high group at the 250‐m and 500‐m buffers.

**TABLE 3:** Stratified analysis of the association between residential greenness and atopic dermatitis in children.

Urbanization level														*p*‐value for interaction
Buffer	NDVI	Metropolitan (*n* = 7454)						Non‐metropolitan (*n* = 7477)						
		Crude model			Adjusted model			Crude model			Adjusted model			
		RR (95% CI)	*p*‐value	*p* for trend	RR (95% CI)	*p*‐value	*p* for trend	RR (95% CI)	*p*‐value	*p* for trend	RR (95% CI)	*p*‐value	*p* for trend	
250 m	Low	Ref		.550	Ref		.**022**	Ref		.**004**	Ref		.**003**	Ref
	Moderate	0.91 (0.77–1.07)	.238		**0.81 (0.67–0.96)**	.**018**		**0.82 (0.70–0.97)**	.**017**		**0.80 (0.67–0.94)**	.**007**		.739
	High	0.95 (0.81–1.12)	.555		**0.79 (0.64–0.96)**	.**019**		**0.79 (0.67–0.93)**	.**004**		**0.74 (0.61–0.91)**	.**004**		.128
500 m	Low	Ref		.946	Ref		.**039**	Ref		.096	Ref		.099	Ref
	Moderate	0.94 (0.80–1.10)	.443		**0.82 (0.69–0.99)**	.**037**		**0.84 (0.71–0.99)**	.**034**		**0.81 (0.68–0.96)**	.**018**		.291
	High	1.01 (0.86–1.18)	.944		**0.80 (0.65–0.98)**	.**034**		0.87 (0.74–1.03)	.096		0.85 (0.69–1.04)	.104		.089
1000 m	Low	Ref		.279	Ref		.370	Ref		.356	Ref		.488	Ref
	Moderate	1.04 (0.88–1.23)	.618		0.96 (0.80–1.14)	.618		0.87 (0.74–1.03)	.106		0.88 (0.74–1.05)	.161		.114
	High	1.09 (0.93–1.28)	.279		0.90 (0.72–1.13)	.371		0.93 (0.79–1.09)	.358		0.93 (0.75–1.16)	.531		.**027**

Child's sex														
Buffer	NDVI	Boy (*n* = 7698)						Girl (*n* = 7234)						*p*‐value for interaction
		Crude model			Adjusted model			Crude model			Adjusted model			
		RR (95% CI)	*p*‐value	*p* for trend	RR (95% CI)	*p*‐value	*p* for trend	RR (95% CI)	*p*‐value	*p* for trend	RR (95% CI)	*p*‐value	*p* for trend	
250 m	Low	Ref		.527	Ref		.**018**	Ref		.**035**	Ref		.259	Ref
	Moderate	1.07 (0.92–1.25)	.365		0.95 (0.80–1.13)	.563		0.87 (0.74–1.03)	.112		0.88 (0.73–1.06)	.183		.071
	High	0.95 (0.81–1.11)	.524		**0.74 (0.59–0.94)**	.**013**		**0.83 (0.70–0.99)**	.**035**		0.88 (0.68–1.13)	.321		.224
500 m	Low	Ref		.860	Ref		.088	Ref		.**031**	Ref		.236	Ref
	Moderate	0.996 (0.85–1.16)	.960		0.90 (0.75–1.07)	.232		0.90 (0.76–1.07)	.242		0.90 (0.75–1.09)	.285		.532
	High	0.99 (0.84–1.15)	.860		0.81 (0.64–1.03)	.091		**0.83 (0.70–0.98)**	.**031**		0.86 (0.66–1.12)	.270		.072
1000 m	Low	Ref		.880	Ref		.078	Ref		.147	Ref		.774	Ref
	Moderate	0.94 (0.80–1.10)	.450		0.85 (0.70–1.02)	.073		1.01 (0.85–1.19)	.926		1.06 (0.88–1.28)	.545		.569
	High	0.99 (0.85–1.15)	.881		0.81 (0.63–1.04)	.100		0.88 (0.74–1.05)	.147		1.03 (0.78–1.36)	.854		.216

Parental allergic diseases														
Buffer	NDVI	Children with parental allergic diseases (*n* = 6122)						Children without parental allergic diseases (*n* = 6328)						*p*‐value for interaction
		Crude model			Adjusted model			Crude model			Adjusted model			
		RR (95% CI)	*p*‐value	*p* for trend	RR (95% CI)	*p*‐value	*p* for trend	RR (95% CI)	*p*‐value	*p* for trend	RR (95% CI)	*p*‐value	*p* for trend	
250 m	Low	Ref		.455	Ref		.638	Ref		.**001**	Ref		.**001**	Ref
	Moderate	1.11 (0.95–1.30)	.191		1.07 (0.90–1.28)	.414		0.87 (0.71–1.07)	.185		**0.78 (0.63–0.98)**	.**032**		.066
	High	1.06 (0.91–1.24)	.456		0.93 (0.74–1.17)	.515		**0.70 (0.57–0.87)**	.**001**		**0.58 (0.42–0.80)**	.**001**		.**007**
500 m	Low	Ref		.289	Ref		.871	Ref		.**018**	Ref		.058	Ref
	Moderate	1.10 (0.94–1.29)	.241		1.07 (0.89–1.27)	.467		0.87 (0.71–1.07)	.175		0.81 (0.64–1.03)	.082		.076
	High	1.09 (0.93–1.27)	.289		0.96 (0.76–1.23)	.760		**0.78 (0.63–0.96)**	.**018**		0.75 (0.54–1.03)	.079		.**022**
1000 m	Low	Ref		.495	Ref		.470	Ref		.118	Ref		.653	Ref
	Moderate	1.06 (0.91–1.24)	.452		1.01 (0.84–1.21)	.943		0.94 (0.76–1.15)	.540		0.93 (0.74–1.17)	.526		.430
	High	1.06 (0.90–1.24)	.495		0.90 (0.70–1.16)	.418		0.84 (0.68–1.04)	.118		0.94 (0.68–1.31)	.718		.117

*Note*: Adjusted model: Adjusted for maternal age at delivery, maternal pre‐pregnancy body mass index, maternal history of allergic diseases, paternal history of allergic diseases, maternal educational attainment, household income, child's sex, season of birth, presence of older siblings, exclusive breastfeeding, passive smoking in children, pet ownership of dogs or cats, use of childcare facilities, NO_2_, PM_2.5_, areal deprivation index, urbanization level, except for the stratification variable in each analysis. Bold: *p* < .05.

Abbreviations: CI, confidence interval; NDVI, normalized difference vegetation index; RR, risk ratio.

## 4 DISCUSSION

The present study examined the association between early‐life exposure to residential greenness and the development of AD for up to the age of 5 years. A significant protective association was observed within the 250‐ and 500‐m buffers. Significant protective associations were also observed in both metropolitan and non‐metropolitan areas, among boys and children without a parental history of allergic diseases. To our knowledge, this is the first study in Japan to demonstrate an association between residential greenness and the development of AD in children.

Previous studies on residential greenness and AD have reported inconsistent findings. Three studies (South Korea and France) have shown associations between higher levels of prenatal greenness and lower risks of AD,^21^, ^22^, ^23^ whereas two studies (China and Finland) observed an increased risk.^24^, ^25^ In addition, studies in China, Portugal, and across Austria and Italy, and nine European cohorts reported no association.^26^, ^27^, ^28^, ^29^ These discrepancies may reflect methodological differences across studies, including exposure assessment, vegetation types, and buffer sizes. In addition, while our study focused on AD, some previous studies used the term eczema. Although the terminology is not fully standardized, eczema may have been used interchangeably with AD in many studies.^40^ Our findings add evidence from a large Japanese birth cohort suggesting a protective association between residential greenness and AD.

With regard to exposure assessment, seven previous studies have used NDVI,^22^, ^23^, ^24^, ^25^, ^27^, ^28^, ^29^ and five of them combined NDVI with other indicators, such as land cover classes, species richness index, or distance to the nearest park. Notably, a study in Finland reported no association between NDVI and atopic eczema but found that coniferous forest was associated with elevated eczema risk. Therefore, future research should not only incorporate NDVI but also information on vegetation composition, including tree species.

Buffer size is another factor that varied across studies. Previous studies examined multiple buffer radii ranging from 100 m to 1250 m. Similarly, in the present study, we evaluated NDVI within 250‐, 500‐, and 1000‐m buffers. When an association was observed in other studies, it tended to appear within smaller buffer zones, consistent with our study findings where the greenness closer to the residential area showed a stronger association with AD.

Furthermore, among previous studies that reported a protective association in children, two evaluated exposure to green environments during pregnancy,^21^, ^22^ and one was a cross‐sectional study.^23^ In contrast, in our study, we examined exposure to green environments during the first 6 months after birth and observed a protective association. Given that greenness during pregnancy is likely to be strongly correlated with greenness after birth, these findings suggest that exposure to greenness from the prenatal through the postnatal period is essential.

Our stratified analyses showed largely homogeneous associations between residential greenness and AD across subgroups, with some heterogeneity. Because the accessibility, type (e.g., parks, street trees, and forests), and composition (e.g., tree species, relative proportions of trees, grass, and shrubs) of greenness differed between urban and rural areas, the association between greenness and allergic diseases has been suggested to depend on the degree of urbanization.^22^, ^30^ Our findings were generally consistent between metropolitan and non‐metropolitan areas, and the point estimates were largely similar despite a statistically significant interaction observed for the 1000‐m buffer. In sensitivity analyses with further stratification of non‐metropolitan areas, the results were largely consistent overall. A positive association between residential greenness within the 1000‐m buffer and AD was observed in large cities and accessible small towns; however, this finding should be interpreted with caution given the small sample size. Taken together, these results suggest potential effect heterogeneity according to the degree of urbanization and highlight the importance of analyses stratified by urbanization level. In analyses stratified by the child's sex, no statistically significant interaction was observed. However, the protective association appeared stronger in boys than in girls, consistent with previous studies.^27^, ^28^ Potential sex differences in early‐life skin barrier function and immune development may cause boys to be more susceptible to environmental exposures, which could explain the stronger association in boys. For example, sex‐, age‐, and body site–related differences in transepidermal water loss rates and stratum corneum hydration, as well as a tendency for Th2‐shifted immune responses in male infants, have been reported.^31^, ^41^ In analyses stratified by parental history of allergic diseases, the protective association of residential greenness was evident only among children without a parental history of allergic diseases, and a statistically significant interaction was observed. Children with a parental history may have a stronger baseline predisposition to AD due to inherited factors. This includes genetic variants affecting skin barrier function, such as mutations in the *filaggrin* gene, which encodes proteins that play key roles in the formation of the skin barrier.^1^ When such predisposition is high, the relative contribution of environmental factors may become less apparent.

Several mechanisms may underlie this association. Greenness provides biologically diverse environments, and microbe‐rich environments play a protective role against the development of allergic diseases.^19^, ^42^ Biodiversity loss has been reported to reduce interactions between environmental and human microbiota, leading to immune dysfunction and impaired tolerance mechanisms in humans.^42^ Atopic individuals have been shown to have lower environmental biodiversity in the surroundings of their homes compared with non‐atopic individuals.^43^ Moreover, residential greenness was associated with a reduction in air pollutants, which has been shown to increase the risk of allergic diseases.^17^, ^44^ Maternal exposure to air pollution during pregnancy increases the risk of infantile AD, and this risk is reduced in residential areas with sufficient green space.^21^ In this study, the association between residential greenness and AD persisted after adjustment for air pollution. Thus, multiple mechanisms may interact to contribute to the pathway linking residential greenness to AD.

From a public health perspective, our findings may help inform strategies for preventing allergic diseases through considerations for green environments in urban planning and residential choices for expectant mothers. The results also suggest that children living in areas with low residential greenness represent a high‐risk group for AD and may benefit from close monitoring of symptoms. Future research should further explore practical preventive approaches, including increasing opportunities for children to spend time in green environments or promoting greenness in residential areas, ultimately aiding the early prevention and management of allergic diseases.

This study had several strengths. It was conducted using data from a large‐scale birth cohort. Moreover, geographic factors, including the degree of urbanization and exposure to air pollution, were controlled. Notably, this is the first study in Japan to investigate the association between residential greenness and AD in children.

Despite its strengths, this study had some limitations. First, it was conducted only in Miyagi Prefecture, Japan, where the composition of tree species and types of greenness may differ from those in other regions, potentially limiting the generalizability of the findings. Second, residential greenness was assessed using NDVI derived from satellite imagery from a single year, which may not fully capture smaller‐scale or more recent changes in greenness, such as new developments or newly created green spaces. Furthermore, although we excluded children who moved within the first 6 months of life to better define early‐life exposure, temporal variations in NDVI and residential mobility may have resulted in exposure misclassification. Third, individual‐level behavioral factors, such as the amount of time children spend outdoors and the nature of their outdoor activities, were not assessed. These factors could have influenced both exposure to greenness and its effects on AD. Fourth, we did not evaluate specific vegetation characteristics, including species diversity or pollen abundance, or potential mediating factors, such as noise, social interaction, and stress; this limited our ability to infer the underlying biological mechanisms. Fifth, the outcome, AD in children, was assessed based on maternal self‐reporting of a physician's diagnosis without clinical validation or standardized diagnostic criteria. This report may have been influenced by parental allergy status or recall bias. In addition, potential diagnostic heterogeneity (including distinctions between eczema and AD) may exist, and information on disease severity, persistence, and number of episodes was not available. Therefore, the findings should be interpreted with caution. Finally, this study focused specifically on AD in children. However, AD often coexist or develop sequentially over time, which is known as the atopic march.^45^ Therefore, further research is needed to examine the associations between greenness and other allergic diseases, such as asthma, allergic rhinitis, and food allergy.

## 5 CONCLUSIONS

This study examined the association between residential greenness during early life and the subsequent development of AD up to the age of 5 years and found a protective association with residential greenness. These findings highlight the potential importance of early‐life residential greenness in the prevention of AD. Findings from this study may support future research in strengthening evidence on the potential health benefits of greenness and contribute to strategies for the early prevention of allergic diseases in children.

## AUTHOR CONTRIBUTIONS

**Ami Uematsu:** Conceptualization; methodology; formal analysis; visualization; writing – original draft. **Hisashi Ohseto:** Conceptualization; methodology; writing – review and editing. **Masatsugu Orui:** Conceptualization; methodology; data curation; investigation; writing – review and editing. **Zheng Xian:** Conceptualization; methodology; writing – review and editing. **Yuta Takahashi:** Conceptualization; methodology; writing – review and editing. **Geng Chen:** Conceptualization; methodology; writing – review and editing. **Keiko Murakami:** Data curation; investigation; writing – review and editing. **Mami Ishikuro:** Data curation; investigation; writing – review and editing. **Aoi Noda:** Data curation; investigation; writing – review and editing. **Genki Shinoda:** Data curation; investigation; writing – review and editing. **Taku Obara:** Data curation; investigation; supervision; writing – review and editing. **Tomoki Nakaya:** Data curation; supervision; writing – review and editing. **Shinichi Kuriyama:** Data curation; investigation; supervision; writing – review and editing.

## FUNDING INFORMATION

The TMM BirThree Cohort Study was supported by grants from the Ministry of Education, Culture, Sports, Science and Technology (MEXT) and Japan Agency for Medical Research and Development (AMED) (Grant Nos. JP21tm0124005, JP21tm0424601, and JP24gn0110088). This study was supported by the JST SPRING (Grant No. JPMJSP2114), Japan Society for the Promotion of Science (JSPS) KAKENHI Grants (Grant No. 25KJ0575), and Endowed Department of Traffic and Medical Informatics in Disaster, Tohoku Medical Megabank Organization, Tohoku University, which is funded through a donation from East Japan Railway Company.

## CONFLICT OF INTEREST STATEMENT

The authors have no conflicts of interest to declare.

## Supporting information


Table S1.

## Data Availability

The data that support the findings of this study are available from the TMM biobank; however, restrictions apply to the availability of these data, which were used under license for the current study and hence are not publicly available. Data are available from the authors upon reasonable request and with the permission of the TMM biobank. All inquiries about data access should be sent to the TMM biobank (dist@megabank.tohoku.ac.jp).
