# Supplementary material for: Residential greenness and atopic dermatitis in Japanese children: Findings from the TMM BirThree cohort study
Source: Pediatr Allergy Immunol. 2026 Apr 6;37(4):e70338. doi: 10.1111/pai.70338 (PMC13053634; doi:10.1111/pai.70338)
Supplement: Supplementary file 1 — Table S1. [file PAI-37-e70338-s001.docx]

**Supplementary Materials**

Table S1. Sensitivity analysis using tertile cutoffs derived from the 250-m buffer applied to other buffer sizes

|  |  | Crude model | | |  | Adjusted model | | |
| --- | --- | --- | --- | --- | --- | --- | --- | --- |
| Buffer | NDVI | RR (95% CI) | P-value | P for trend |  | RR (95% CI) | P-value | P for trend |
| 250 m | Low | Ref |  | 0.054 |  | Ref |  | **0.014** |
|  | Moderate | 0.99 (0.88–1.11) | 0.871 |  |  | 0.94 (0.83–1.06) | 0.318 |  |
|  | High | 0.89 (0.79–1.00) | 0.054 |  |  | **0.80 (0.67–0.95)** | **0.012** |  |
| 500 m | Low | Ref |  | 0.081 |  | Ref |  | **0.019** |
|  | Moderate | 1.08 (0.95–1.22) | 0.241 |  |  | 1.02 (0.89–1.16) | 0.798 |  |
|  | High | 0.92 (0.82–1.03) | 0.132 |  |  | **0.81 (0.69–0.96)** | **0.015** |  |
| 1000 m | Low | Ref |  | 0.088 |  | Ref |  | **0.032** |
|  | Moderate | 1.11 (0.97–1.28) | 0.138 |  |  | 1.05 (0.91–1.22) | 0.494 |  |
|  | High | 0.94 (0.83–1.08) | 0.393 |  |  | 0.85 (0.71–1.02) | 0.074 |  |

Tertiles of NDVI derived from the 250‑m buffer were defined as low (<0.097), moderate (0.097–0.136), and high ($\geq$0.136).

CI, confidence interval; NDVI, normalized difference vegetation index; RR, risk ratio

Bold: p < 0.05.

Adjusted model: Adjusted for maternal age at delivery, maternal pre-pregnancy body mass index, maternal history of allergic diseases, paternal history of allergic diseases, maternal educational attainment, household income, child’s sex, season of birth, presence of older siblings, exclusive breastfeeding, passive smoking in children, pet ownership of dogs or cats, use of childcare facilities, NO_2_, PM_2.5_, areal deprivation index, urbanization level

Table S2. Stratified analysis by subcategories of urbanization level of the association between residential greenness and atopic dermatitis in children

|  |  | Metropolitan (n = 7,454) | | | | | | |
| --- | --- | --- | --- | --- | --- | --- | --- | --- |
|  |  | Crude model | | |  | Adjusted model | | |
| Buffer | NDVI | RR (95% CI) | P-value | P for trend |  | RR (95% CI) | P-value | P for trend |
| 250 m | Low | Ref |  | 0.550 |  | Ref |  | **0.022** |
|  | Moderate | 0.91 (0.77–1.07) | 0.238 |  |  | **0.81 (0.67–0.96)** | **0.018** |  |
|  | High | 0.95 (0.81–1.12) | 0.555 |  |  | **0.79 (0.64–0.96)** | **0.019** |  |
| 500 m | Low | Ref |  | 0.946 |  | Ref |  | **0.039** |
|  | Moderate | 0.94 (0.80–1.10) | 0.443 |  |  | **0.82 (0.69–0.99)** | **0.037** |  |
|  | High | 1.01 (0.86–1.18) | 0.944 |  |  | **0.80 (0.65–0.98)** | **0.034** |  |
| 1000 m | Low | Ref |  | 0.279 |  | Ref |  | 0.370 |
|  | Moderate | 1.04 (0.88–1.23) | 0.618 |  |  | 0.96 (0.80–1.14) | 0.618 |  |
|  | High | 1.09 (0.93–1.28) | 0.279 |  |  | 0.90 (0.72–1.13) | 0.371 |  |
|  |  | Large cities and accessible small towns (n = 974) | | | | | | |
|  |  | Crude model | | |  | Adjusted model | | |
| Buffer | NDVI | RR (95% CI) | P-value | P for trend |  | RR (95% CI) | P-value | P for trend |
| 250 m | Low | Ref |  | 0.545 |  | Ref |  | 0.673 |
|  | Moderate | 1.30 (0.82-2.07) | 0.262 |  |  | 1.35 (0.85-2.14) | 0.208 |  |
|  | High | 0.85 (0.51-1.42) | 0.527 |  |  | 0.86 (0.48-1.53) | 0.597 |  |
| 500 m | Low | Ref |  | 0.280 |  | Ref |  | 0.405 |
|  | Moderate | 1.02 (0.65-1.63) | 0.917 |  |  | 1.05 (0.58-1.89) | 0.868 |  |
|  | High | 0.76 (0.46-1.26) | 0.281 |  |  | 0.78 (0.38-1.59) | 0.499 |  |
| 1000 m | Low | Ref |  | 0.107 |  | Ref |  | **0.006** |
|  | Moderate | 1.39 (0.83-2.32) | 0.206 |  |  | **1.74 (1.01-3.00)** | **0.046** |  |
|  | High | 1.50 (0.91-2.47) | 0.111 |  |  | **2.40 (1.28-4.52)** | **0.006** |  |
|  |  | Remote small towns (n = 1,574) | | | | | | |
|  |  | Crude model | | |  | Adjusted model | | |
| Buffer | NDVI | RR (95% CI) | P-value | P for trend |  | RR (95% CI) | P-value | P for trend |
| 250 m | Low | Ref |  | **0.018** |  | Ref |  | 0.058 |
|  | Moderate | 1.00 (0.73–1.38) | 0.999 |  |  | 0.99 (0.68–1.44) | 0.952 |  |
|  | High | **0.64 (0.44–0.93)** | **0.019** |  |  | 0.65 (0.41–1.03) | 0.066 |  |
| 500 m | Low | Ref |  | **0.004** |  | Ref |  | **0.021** |
|  | Moderate | 0.91 (0.66–1.25) | 0.570 |  |  | 0.95 (0.65–1.38) | 0.779 |  |
|  | High | **0.58 (0.40–0.85)** | **0.005** |  |  | **0.62 (0.40–0.94)** | **0.026** |  |
| 1000 m | Low | Ref |  | **0.040** |  | Ref |  | 0.301 |
|  | Moderate | 1.05 (0.76–1.45) | 0.760 |  |  | 1.09 (0.76–1.56) | 0.629 |  |
|  | High | **0.68 (0.47–0.98)** | **0.041** |  |  | 0.75 (0.47–1.20) | 0.237 |  |
|  |  | Accessible rural settlements (n = 1,438) | | | | | | |
|  |  | Crude model | | |  | Adjusted model | | |
| Buffer | NDVI | RR (95% CI) | P-value | P for trend |  | RR (95% CI) | P-value | P for trend |
| 250 m | Low | Ref |  | 0.550 |  | Ref |  | 0.513 |
|  | Moderate | 0.86 (0.58–1.26) | 0.440 |  |  | 0.75 (0.50–1.10) | 0.141 |  |
|  | High | 1.12 (0.78–1.60) | 0.546 |  |  | 0.86 (0.57–1.30) | 0.471 |  |
| 500 m | Low | Ref |  | 0.840 |  | Ref |  | **0.046** |
|  | Moderate | 1.04 (0.72–1.50) | 0.825 |  |  | 0.66 (0.42–1.04) | 0.076 |  |
|  | High | 0.96 (0.66–1.40) | 0.840 |  |  | **0.59 (0.37–0.96)** | **0.032** |  |
| 1000 m | Low | Ref |  | 0.577 |  | Ref |  | 0.348 |
|  | Moderate | 0.98 (0.68–1.43) | 0.932 |  |  | 0.80 (0.53–1.21) | 0.290 |  |
|  | High | 1.11 (0.77–1.61) | 0.573 |  |  | 0.78 (0.48–1.28) | 0.331 |  |
|  |  | Remote rural settlements (n = 3,491) | | | | | | |
|  |  | Crude model | | |  | Adjusted model | | |
| Buffer | NDVI | RR (95% CI) | P-value | P for trend |  | RR (95% CI) | P-value | P for trend |
| 250 m | Low | Ref |  | 0.094 |  | Ref |  | 0.094 |
|  | Moderate | 0.98 (0.77–1.24) | 0.861 |  |  | 0.93 (0.73–1.19) | 0.561 |  |
|  | High | 0.81 (0.63–1.04) | 0.095 |  |  | 0.78 (0.59–1.04) | 0.090 |  |
| 500 m | Low | Ref |  | 0.425 |  | Ref |  | 0.549 |
|  | Moderate | 1.04 (0.81–1.32) | 0.772 |  |  | 1.02 (0.79–1.30) | 0.902 |  |
|  | High | 0.90 (0.70–1.16) | 0.424 |  |  | 0.90 (0.67–1.22) | 0.509 |  |
| 1000 m | Low | Ref |  | 0.739 |  | Ref |  | 0.997 |
|  | Moderate | 1.07 (0.84–1.37) | 0.563 |  |  | 1.13 (0.88–1.45) | 0.355 |  |
|  | High | 0.96 (0.75–1.23) | 0.739 |  |  | 0.98 (0.70–1.35) | 0.880 |  |

CI, confidence interval; NDVI, normalized difference vegetation index; RR, risk ratio

Bold: p < 0.05.

Adjusted model: Adjusted for maternal age at delivery, maternal pre-pregnancy body mass index, maternal history of allergic diseases, paternal history of allergic diseases, maternal educational attainment, household income, child’s sex, season of birth, presence of older siblings, exclusive breastfeeding, passive smoking in children, pet ownership of dogs or cats, use of childcare facilities, NO_2_, PM_2.5_, areal deprivation index
